# High-performance and recyclable epoxy resins based on fully bio-based hardeners containing Schiff base structures

**DOI:** 10.1098/rsos.250109

**Published:** 2025-07-09

**Authors:** Ruize Zhang, Jiaqi Zhong, Gaosheng Wu, Kaidi Xu, Xiaoli Xu, Xuliang Cui, Baoyun Xu, Xiaolei li

**Affiliations:** ^1^ Shanghai Research Institute of Chemical Industry, Shanghai, People’s Republic of China

**Keywords:** epoxy curing agent, fully bio-based, degradable, Schiff base, vanillin, amino acid

## Abstract

The development of recyclable bio-based epoxy resins has been the focus of research nowadays, particularly in light of increasing environmental concerns and the drive for resource recycling. Herein, two fully bio-based epoxy curing agents, VLYS (fully bio-based Schiff basic epoxy curing agent based on vanillin and lysine) and VLEV (fully bio-based Schiff basic epoxy curing agent based on vanillin and levodopa), were synthesized by reacting amino acids—lysine and levodopa, respectively—with vanillin. Both epoxy resins cured by these two hardeners, VLYS-E and VLEV-E, show high glass transition temperatures (*T*_g_ > 141°C) and superior mechanical properties (Young’s modulus >1000 MPa and flexural modulus >2900 MPa), which are far better than the resin system based on a petroleum-based curing agent. The incorporation of the Schiff base endows these resins with degradability and reprocessability, while exhibiting high-percentage retention of thermal and mechanical properties after two cycles. Taken together, these findings suggest that these fully bio-based curing agents hold significant potential to supplant conventional hardeners.

## 1. Introduction

Epoxy resins are extensively used in the manufacture of composites, electronic packaging, coatings, adhesives and paints, thanks to their exceptional mechanical strength, thermodynamic properties, electrochemical stability and resistance to corrosion [1,2]. A crucial component of epoxy resin is the curing agent, which significantly influents both the performance and the manufacturing process of the resin [3,4]. However, the non-renewability of epoxy curing agents poses a significant challenge. Traditionally, epoxy hardeners are derived from petroleum, a non-renewable resource. The extraction and refining of crude oil not only contribute to the depletion of these finite resources but also exacerbate the emission of greenhouse gases [5]. More critically, most petroleum-based formulations release volatile organic compounds (VOCs) during curing, posing health risks including acute toxicity and potential carcinogenicity [3]. Consequently, there has been a growing interest in the development of bio-based alternatives to petroleum-derived curing agents. Various bio-based resources, including vegetable oils [6], cardanol [7], colophony [8], gallic acid [9], amino acids and lignin [10], have been explored for their potential in creating renewable epoxy thermosets. Among these, epoxy resins derived from vegetable oils and cardanol tend to exhibit reduced thermal and mechanical properties [11,12]. Meanwhile, the production capacities of colophony and gallic acid are not yet sufficient to meet the demand of large-scale industrial applications [13]. Epoxy resin cured with amino acids or their derivatives generally exhibit poor solvent resistance and unsatisfactory thermomechanical properties. Reported *T*_g_ of common amino acid cured resins is typically below 130°C [14,15].

Conventional epoxy resins also face another great challenge: their permanent cross-linking structure prevents degradation and reprocessing, leading to substantial environmental pollution and resource waste owing to the generation of epoxy waste during production and after its service life [2,16,17]. To address this issue, the introduction of dynamic covalent bonds into epoxy resins has emerged as a promising solution for achieving degradability and recyclability. Among these dynamic bonds, the imine bond (Schiff base) stands out as a particularly promising approach to confer recyclability upon epoxy copolymers [18]. This is because the imine bond can participate in three dynamic reactions (imine exchange, imine metathesis and imine hydrolysis) without the need for any catalyst, simplifying the reprocessing process [19]. Rashid *et al.* [20] developed a bio-based imine hardener (VIH) from vanillin and dimethylenediamine, which enables the cured epoxy vitrimer to be hydrolysed under acidic conditions or reprocessed through hot pressing after crushing. Notably, the reprocessed resin maintained a low percentage deterioration in mechanical properties. Despite its polyaromatic structure, the *T*_g_ of this vitrimer is 93°C. Memon *et al.* [21] prepared another imine curing agent (IH-VAN (Imine-Containing Hardener Based on Vanillin)) using vanillin and isophorone diamine (IPDA), which allowed the cured epoxy vitrimer to be reprocessed either directly through hot pressing or after degradation in an IPDA solution. Similar to the previous example, the *T*_g_ of the vitrimer was also below 120℃. However, the thermal performance of epoxy vitrimers based on these bio-based curing agents is generally lower than that of traditional epoxy resins, which may limit their application. Additionally, these bio-based hardeners contain non-biodegradable amines, indicating a need for further enhancement of the bio-based components to improve their environmental sustainability.

Based on our limited knowledge, fully bio-based curing agents are rarely reported. There is a pressing need to develop epoxy vitrimers that not only offer superior performance but are also straightforward to prepare and exhibit exceptional recyclability. This research has made progress in this direction by synthesizing two fully bio-based epoxy curing agents, VLYS (fully bio-based Schiff basic epoxy curing agent based on vanillin and lysine) and VLEV (fully bio-based Schiff basic epoxy curing agent based on vanillin and levodopa), which incorporated imine bonds. These curing agents were derived from vanillin, a bio-based aldehyde sourced from the oxidation of lignin, renowned for its benzene rings and multiple reactive sites [22]. Additionally, the use of l-lysine and levodopa, both of which were naturally occurring amines with a plethora of functional groups, enabled the curing of epoxy monomers [23]. The resulting epoxy vitrimers, VLYS-E and VLEV-E, demonstrated remarkable properties such as a high *T*_g_ exceeding 140°C, along with impressive mechanical characteristics, including Young’s modulus and bending modulus. These vitrimers also showed an enviable capacity for degradation in acidic environments, allowing for reprocessing through hot pressing. Notably, even after two cycles of reprocessing, these materials retained their desirable mechanical performance, underscoring their potential for sustainable applications.

## 2. Material and methods

### 2.1. Materials

Vanillin (VAN), l-lysine (LYS, 98%), levodopa (LEV, 98%), 4,4′-diaminodiphenyl methane (DDM), 1,2-dimethylimidazole and imidazole were purchased from Aladdin Biochemical Technology Co., Ltd. Diglycidyl ether of bisphenol A (DGEBA) was supplied by Hangzhou Wuhuigang Adhesive Co., methanol, ethanol, diethyl ether, hydrochloric acid (37%) and sodium hydroxide were obtained from Sinopharm Chemical Reagent Co., Ltd. All the chemicals were used as received.

### 2.2. Synthesis of fully bio-based epoxy curing agents

#### 2.2.1. Synthesis of VLYS

LYS (0.1 mol, 14.6 g) and 100 ml anhydrous ethanol were added to a three-necked flask equipped with a reflux condenser under nitrogen atmosphere. VAN (0.1 mol, 15.2 g) was dissolved in 100 ml ethanol and the dissolved solution was added dropwise with vigorous stirring within 2 h. After the drop was completed, the mixture was heated to 60℃ and continued for 5 h. Subsequently, the reaction system was cooled to room temperature and then filtered, and the filtration residue was washed (three times) using ethanol. After that, the solid product was dried under vacuum at 60°C for 24 h, and the yellow powder VLYS was obtained (17.1 g, yield = 86%).

#### 2.2.2. Synthesis of VLEV

The synthesis of VLEV was similar to VLYS except that l-lysine was replaced by levodopa and sodium hydroxide was added additionally in equimolar to levodopa. After the reaction was complete, the system was cooled to room temperature. The brownish yellow precipitate obtained by filtration was dissolved in 100 ml distilled water and pH was adjusted to 5 using 5 wt% hydrochloric acid. Then the mixture was filtered, and the filtration residue was washed (three times) with distilled water. After that, the solid product was dried under vacuum at 80°C for 24 h, and the yellow powder VLYS was obtained (15.6 g, yield = 84%).

### 2.3. Curing of epoxy resins

VLYS-E, VLEV-E and DDM-E were prepared by curing DGEBA with VLYS, VLEV and DDM. The curing process was divided into two stages: pre-curing and post-curing.

#### 2.3.1. Pre-curing of VLYS-E

This initial stage involves blending DGEBA (30 g) with VLYS (10.7 g) and curing accelerators (imidazole and 1,2-dimethylimidazole, *m*_imidazole_ = *m*_1,2-dimethylimidazole_ = 0.2 g). The mixture is then stirred at 80℃ for 30 min. The molar ratio of epoxy groups in DGEBA to VLYS was maintained at 4 : 1 to ensure optimal cross-linking. Following this, the pre-cured mixture was carefully poured into a suitably sized metal mould and subjected to vacuum defoaming for 30 min.

#### 2.3.2. Post-curing of the epoxy resin

This involved heating the resin under specific conditions to complete the curing reaction. It was carried out at the following conditions: 90°C for 2 h, 150°C for 2 h and 190°C for 2 h. The curing conditions, including those for VLEV-E and DDM-E, were determined through non-isothermal cure kinetics analysis based on differential scanning calorimetry (DSC).

### 2.4. Characterization

#### 2.4.1. Fourier transform infrared

Fourier transform infrared (FT-IR) spectra were recorded on a PerkinElmer Spectrum II in the 400−4000 cm^−1^ region using KBr (potassium bromide) pellets.

#### 2.4.2. Nuclear magnetic resonance

The ^1^H and ^13^C nuclear magnetic resonance (NMR) spectra were registered on a JNM-ECZ 500 MHz, in dimethylsulfoxide (DMSO)-*d*_6_. Chemical shifts are reported relative to internal DMS (δ 2.5 for ^1^H, δ 40 for ^13^C).

#### 2.4.3. Differential scanning calorimetry

DSC was recorded on a TA Discovery 2500, using N_2_ as a purge gas with the flow rate of 50 ml min^−1^. The curing mixtures were heated from 50 to 300°C at different rates (5, 10, 15 and 20°C min^−1^).

#### 2.4.4. Dynamic mechanical analysis

The dynamic mechanical analysis (DMA) was carried out using a PerkinElmer DMA8000 in double cantilever mode at a frequency of 1 Hz, and the strain of the specimens was fixed at 1% throughout the test. The development of storage modulus (*E*′) and tan δ with temperature were tested for the epoxy resin (20 × 4 × 0.25 mm) in the range of 30−210°C (3°C min^−1^). The *T*_g_ were obtained from the peak of the tan δ curves.

#### 2.4.5. Thermogravimetric analysis

Thermogravimetric analysis (TGA) was recorded on a TG 209F3 instrument under a nitrogen atmosphere (20 ml min^−1^) over a temperature range of 30−800°C at a heating rate of 10°C min^−1^.

#### 2.4.6. Tensile and flexural testing

Tensile and flexural properties were analysed on an Instron-5967 universal testing instrument, following ASTM D638-14 and ASTM D790-17, respectively. The final results reported for each sample was the average of five measurements.

#### 2.4.7. Degradable properties

A weight of 0.5 g of epoxy vitrimer was immersed in acidic solution of 1 M HCl (H_2_O/tetrahydrofuran (v*/*v) = 2 : 8) at 50℃ and the degradation time was recorded.

#### 2.4.8. Self-healing experiment

The VLYS-E was pulverized into a fine powder and subsequently subjected to compression moulding at 190°C and 20 MPa for 60 min, using a stainless steel stamping die (100 × 100 × 1 mm). Once the moulds had been cooled to room temperature, the reprocessed epoxy vitrimer was carefully demoulded and then cut into the required shapes for performance evaluations. The recirculation temperature of VLEV-E was 220 °C, and other conditions and steps were consistent with VLYS-E.

#### 2.4.9. Gel contents

The gel content test of cured epoxy resins was conducted by Soxhlet extraction for 24 h using acetone as the solvent. The insoluble gel fraction was dried under vacuum for 24 h at 70°C and weighed to calculate the gel content. The gel content was calculated by equation (2.1):


(2.1) $$gel\;content=(m/m_{0})\times 100\%,$$

where *m* is the weight of the dry sample and *m*_0_ is the weight of the original sample.

## 3. Results

### 3.1. Synthesis and characterization of VLYS and VLEV

**Scheme 1.**
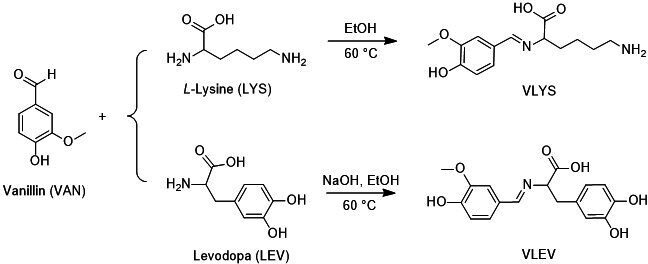
The synthetic route of fully bio-based epoxy curing agents VLYS and VLEV.

The fully bio-based Schiff base epoxy curing agents, VLYS and VLEV (scheme 1), were synthesized through a one-step reaction involving aldehyde-amine condensation, a process that is not only eco-friendly but also straightforward and well-suited for industrial-scale production. The chemical structures of these curing agents were meticulously characterized using FT-IR and NMR spectroscopy.

**Figure 1.**
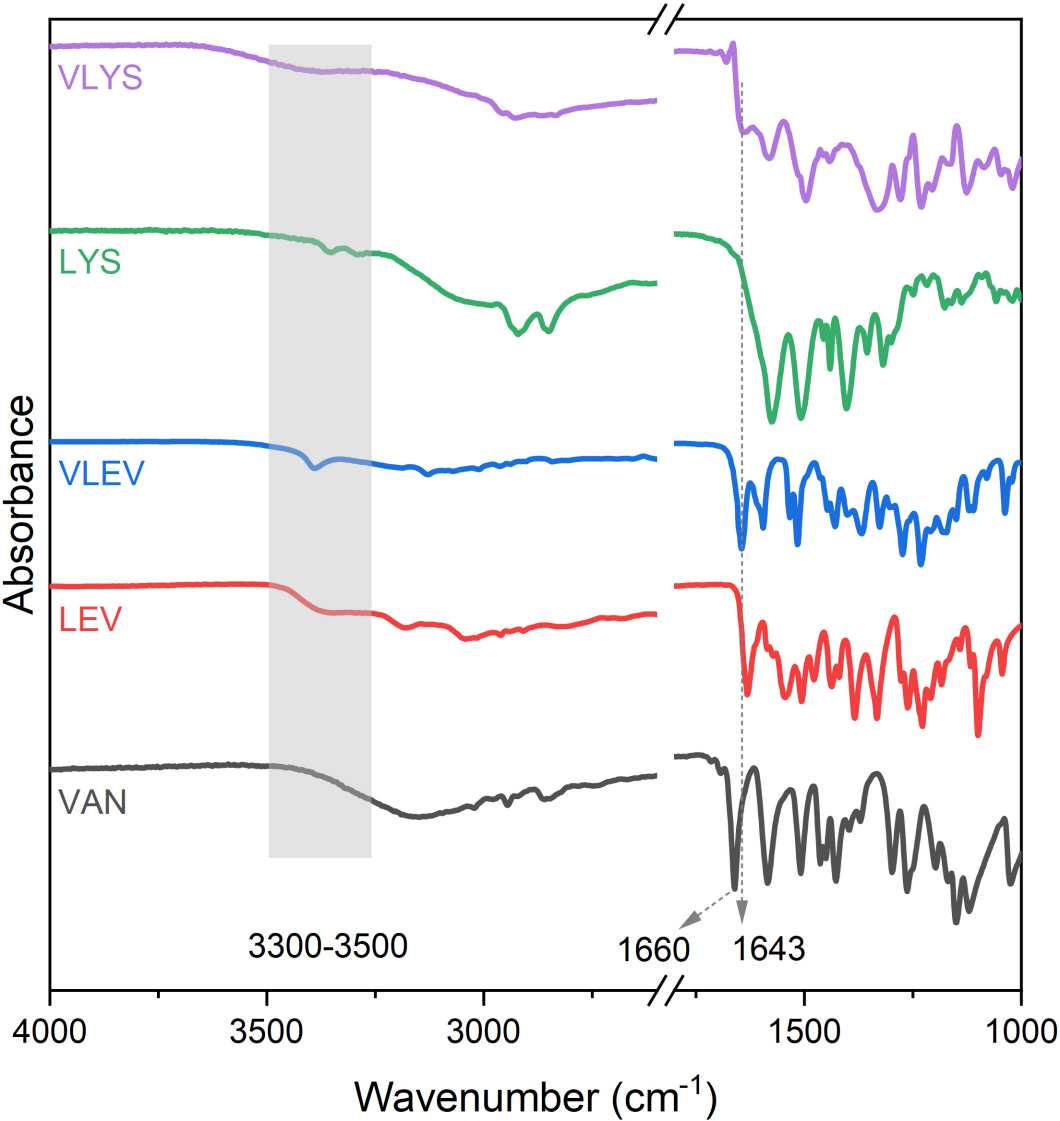
FT-IR spectra of VLYS, LYS, VLEV, LEV and VAN.

In the FT-IR spectra (figure 1), the distinctive imine bond peak was detected at 1643 cm^−1^ for both VLYS and VLEV, and the peak ascribed to the aldehyde group disappeared at 1660 cm^−1^, indicating the successful formation of the Schiff base. After the condensation with LYS and VAN, VLYS exhibited a phenolic group from VAN, in addition to the primary amine. The broad peak ranging from 3300 to 3500 cm^−1^ was attributed to the coupling of N–H and phenolic hydroxyl group (O–H) stretching vibrations. For VLEV, the sharp peak within the same range was owing to the stretching vibration of phenolic groups, as the primary amine in LEV was engaged in Schiff base formation. This analysis demonstrates the successful synthesis of two new compounds containing imine bonds through aldol-amine condensation between the primary amine group of the amino acids and the aldehyde group of vanillin.

**Figure 2.**
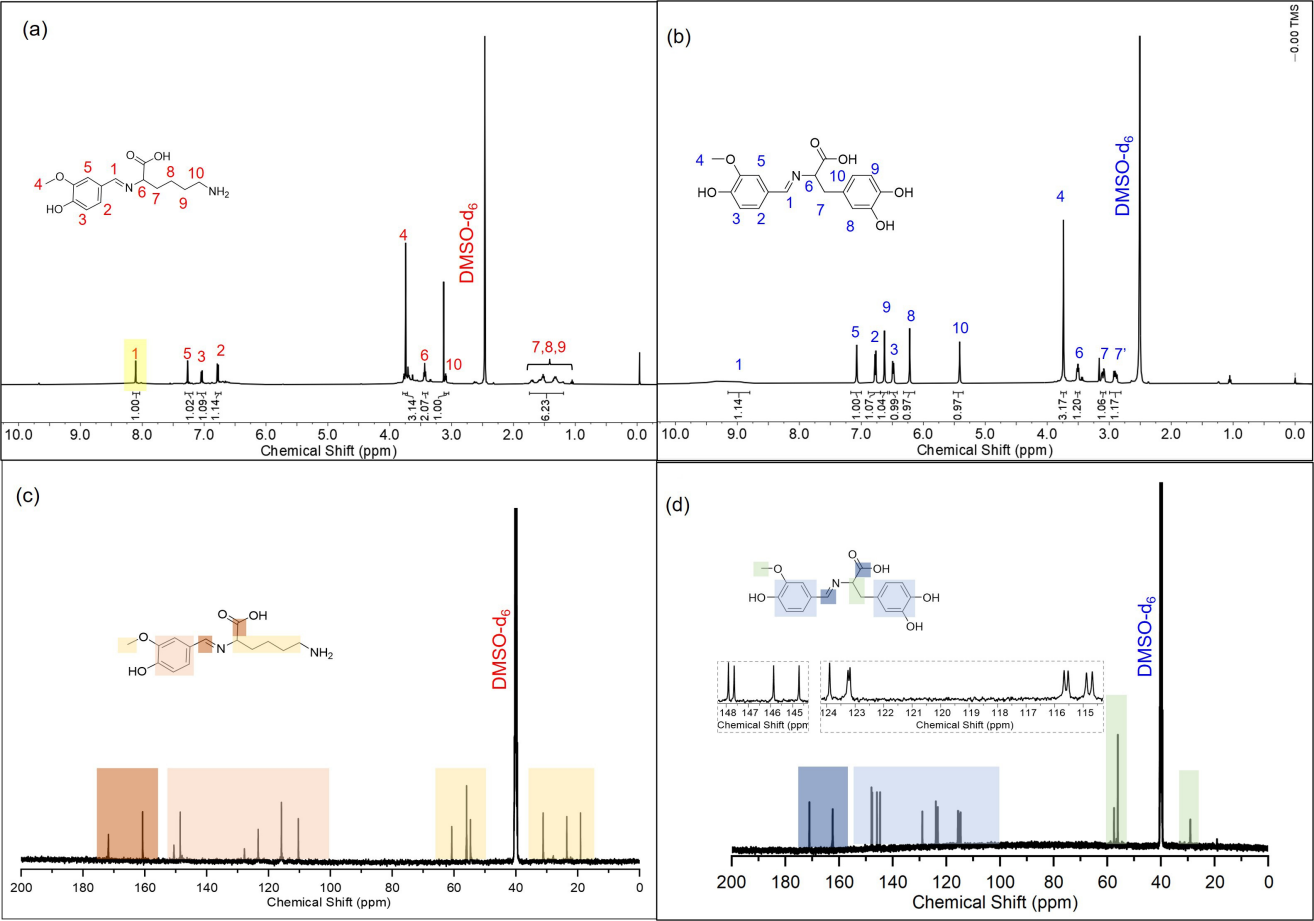
^1^H NMR (a,b) and ^13^C NMR (c,d) spectra of VLYS and VLEV.

In the ^1^H NMR spectra of VLYS and VLEV (figure 2a,b), resonance signals for all protons, except for active hydrogens, were observed. The signal at 9.29 ppm corresponds to the proton in the -CH = N- group, while the proton signals for benzene, methoxy and alkane were found at 6.4–7.4, 3.7 and 1.3–3.6 ppm, respectively. The amine bond peak in VLEV at 9.08 ppm is overshadowed by the phenolic peak, with other proton peaks showing similar chemical shifts to those in VLYS. Furthermore, characterization by ^13^C NMR spectroscopy (figure 2c,d) revealed peaks at 170.0–172.0 and 160.0–162.0 ppm, which were attributed to the carbon atoms in the carboxyl and imine groups, respectively. The carbon resonances for benzene and alkane are also in agreement with the expected chemical structures of the compounds.

All this evidence indicated that VLYS and VLEV were prepared successfully.

### 3.2. Curing behaviours

**Figure 3.**
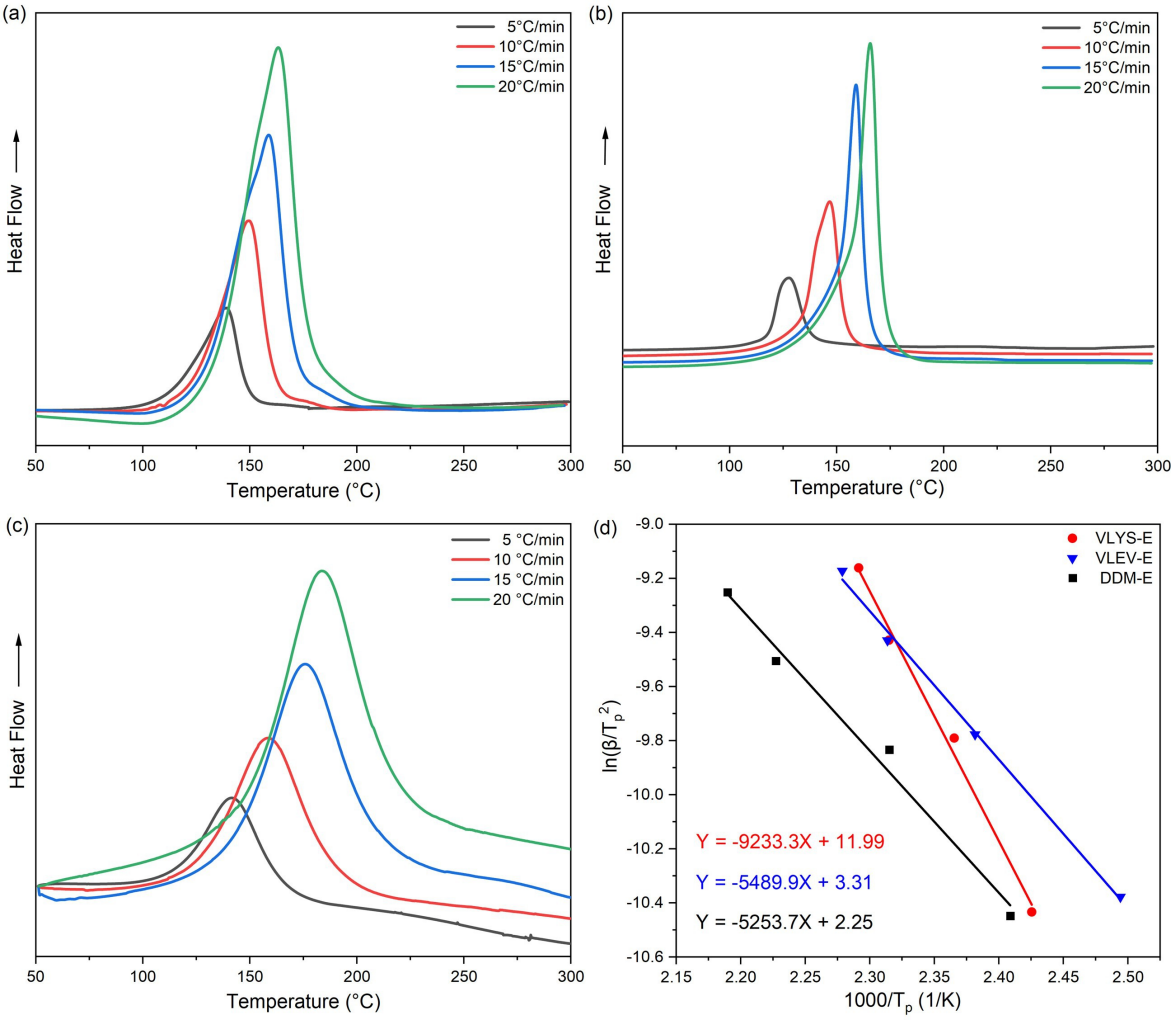
DSC curves of VLYS-E (a), VLEV-E (b), and DDM-E (c) at different heating rates. (d) Linear fitting diagram of ln(β/*T*_p_^2^) versus 1000/*T*_p_ for VLYS-E, VLEV-E and DDM-E.

**Scheme 2.**
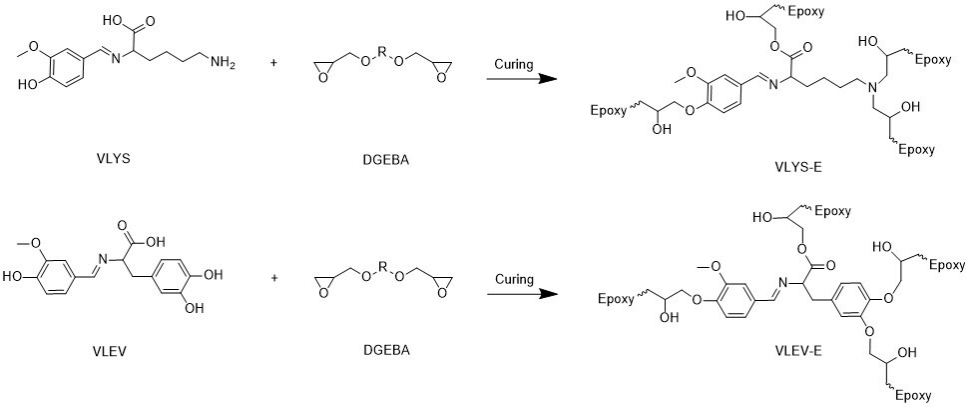
Schematic representation of the curing reaction between fully biobased hardeners and epoxy oligomer illustration.

The active hydrogens present in epoxy curing agents, such as amines, phenols, carboxylic acids and thiols, can initiate the ring-opening reaction of epoxy groups. VLYS-E, contained one primary amine, one phenol and one carboxyl group, while VLEV-E contained three phenol groups and one carboxyl group. Both compounds exhibit a quadruple functionality for curing epoxy monomers (scheme 2). However, owing to the relatively low curing activity of phenolic and carboxyl groups, imidazole and 1,2-dimethylimidazole were incorporated to enhance the curing reaction. To determine the curing conditions and activity of each epoxy resin, non-isothermal curing kinetics were investigated using DSC for VLYS-E, VLEV-E and DDM-E (figure 3). It was shown that curves of each system display a single exothermic peak, suggesting that all three systems were relatively homogeneous solidification systems undergoing a single kinetic process (figure 3a–c). The effective activation energy (*E*_a_) was a crucial parameter for assessing the difficulty of the curing process and can be calculated by the Kissinger equation (equation 3.1):


(3.1) $$\ln\left(\frac{\beta }{T_{p}^{2}}\right)\;=\;-\;\frac{E_{a}}{RT_{p}}\;+\ln\left(\frac{AR}{E_{a}}\right),$$

where *T*_p_ is the exothermic peak temperature, *β* is the heating rate, *A* is the pre-exponential factor and *R* is the ideal gas constant (8.314 J mol^−1^ K^−1^). The *E*_a_ of VLEV-E (45.64 kJ mol^−1^) was found to be comparable to that of DDM-E (43.68 kJ mol^−1^). However, the *E*_a_ for VLYS-E (76.77 kJ mol^−1^) was notably higher (table 1). This increase in Ea for VLYS-E was primarily attributed to the inhibitory effect of carboxyl groups on the reactivity between primary amines and epoxy groups [24]. The characteristic temperature at *β* = 0 for each curing system was determined by performing a linear fit (electronic supplementary material, figure S1) on the data obtained at various *β*-values from the non-isothermal curing exothermic curves measured by DSC (electronic supplementary material, table S1) [25]. Based on these derived characteristic temperatures, the available curing processes for each system were obtained. The curing processes for VLYS-E were 75°C, 2 h + 135°C, 2 h + 205°C, 2 h. The curing conditions for VLEV-E and DDM-E could be determined in the same way, and the curing temperatures for each system are detailed in the electronic supplementary material, table S2. The gel contents of the three epoxy resins (VLYS-E, VLEV-E and DDM-E) were measured at 99.5%, 99.8% and 99.7% (electronic supplementary material, figure S2), respectively, under the given curing conditions. These results indicate that all three systems were fully cured, confirming the feasibility of the applied curing conditions.

**Table 1.** VLYS-E and VLEV-E feature parameters obtained through linear fitting of ln (β/*T*_p_2) and 1000/*T*_p_.

sample	*E*_a_ (kJ mol^−1^)	*R* ^2^
VLYS-E	76.77	0.993
VLEV-E	45.64	0.997
DDM-E	43.68	0.986

### 3.3. Thermodynamic and thermal stability analysis of VLYS-E and VLEV-E

**Figure 4.**
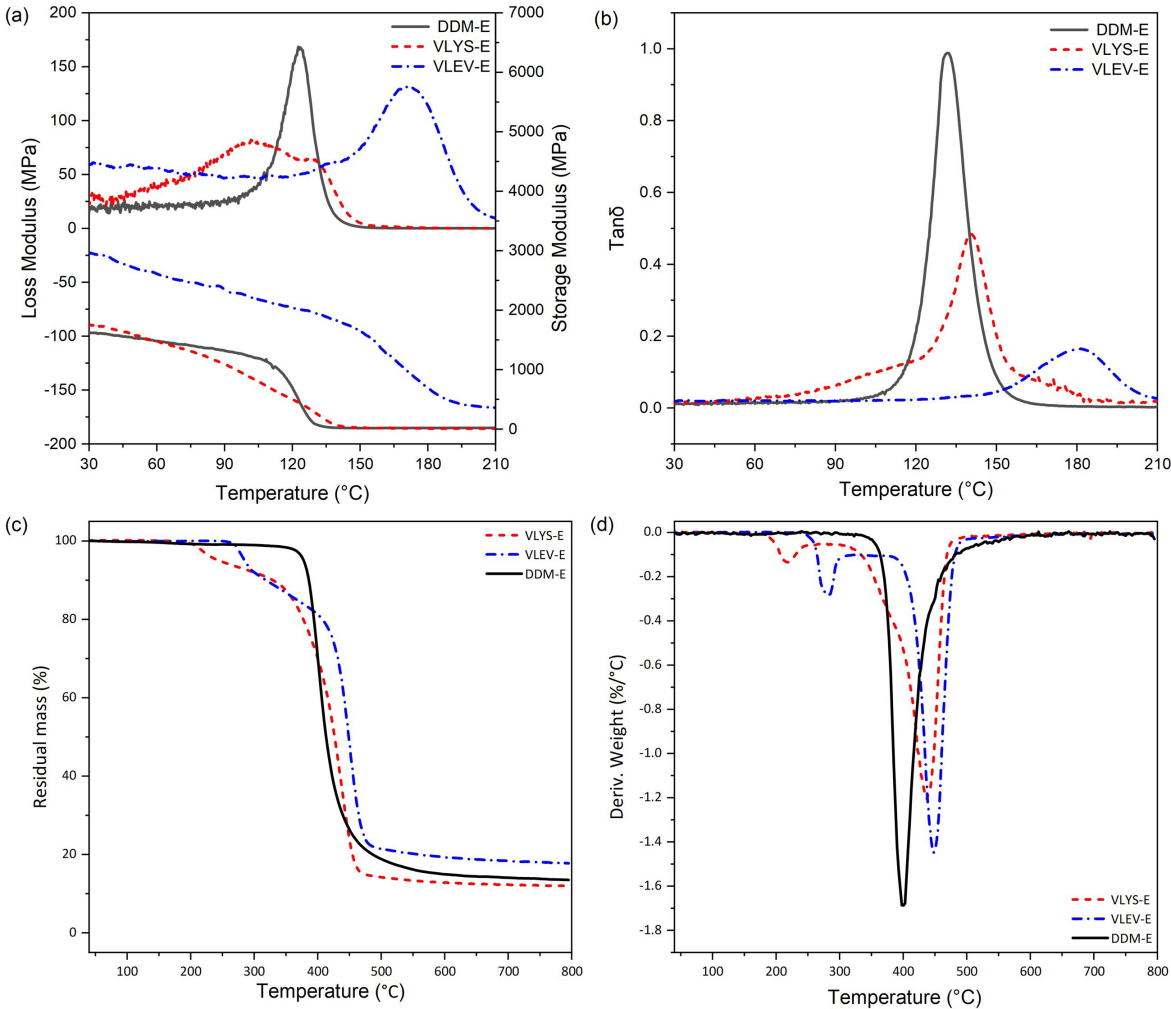
(a) Loss modulus, storage modulus and (b) tan δ versus temperature curves of VLYS-E, VLEV-E and DDM-E, (c) TGA and (d) DTG curves of VLYS-E, VLEV-E and DDM-E with a heating rate of 20 K min^−1^ under N_2_ atmosphere.

The thermo-mechanical properties of VLYS-E and VLEV-E, such as storage modulus (*E*′), loss modulus (E″) and *T*_g_, were investigated by DMA. The storage modulus (*E*′) served as a key indicator of the elastic characteristics of polymers [26]. At 30℃, within the glassy state, the *E*′ (1744 MPa) of VLYS-E was 7.5% higher than DDM-E (1623 MPa). Across all tested temperature ranges, VLEV-E demonstrated a higher storage modulus than DDM-E, reaching 2959 MPa at 30℃—a significant increase of 82.3% compared to DDM-E (figure 4a). These data confirmed that the resins developed in this study possess greater rigidity than the comparative sample, DDM-E. *E*″ reflects the energy dissipated as heat during dynamic deformation. Both VLYS-E and VLEV-E have lower *E*″ peaks than DDM-E. Specifically, the *E*″ of VLYS-E (82 MPa) is less than half of DDM-E (168 MPa), while the peak of VLEV-E occurs at a much higher temperature than DDM-E (figure 4a). The ratio of loss modulus to storage modulus, denoted as tan δ, has its peak corresponding to the resin’s *T*_g_. *T*_g_ was the upper temperature for thermosets, positively correlated with the structural stiffness and crosslink density (*υ*_e_) [27]. The crosslink density can be derived from equation (3.2):


(3.2) $$\upsilon_{e}=\frac{E_{r}}{3RT_{r}},$$

where *T*_r_ is the characteristic temperature, defined as *T*_g_ + 40℃, and *E*_r_ is the storage modulus of the resin at *T*_r_. As shown in figure 4a and table 2, the *υ*_e_ of VLYS-E (1236 mol m^-3^) was lower than DDM-E (1531 mol m^-3^). Unexpectedly, the *T*_g_ of VLYS-E (141℃) was higher than DDM-E (132℃). This discrepancy can be attributed to two factors. Firstly, the branch-like distribution of cross-linking sites within VLYS restricted the movement of the polymer chains. Secondly, the potential reaction between hydroxyl and carboxyl groups within the system to form ester bonds further contributes to the increased rigidity of the structure [15]. The *T*_g_ of VLEV-E was 171℃, which was a 29.5% increase compared to DDM-E (figure 4a). The rigid structure of VLEV was analogous to DDM, and the *υ*_e_ of VLEV-E (29572 mol m^−3^) was significantly higher than DDM-E (1531 mol m^−3^). Consequently, the elevated *T*_g_ of VLEV-E was attributed to the combined effects of its high stiffness and *υ*_e_. In addition, the thermal stability of the two resins derived from fully bio-based curing agents was evaluated by TGA (figure 4c,d). The thermal stability of cured epoxy resins and epoxy composites can be quantified by the statistic heat-resistant index (*T*_s_) [28], which was calculated through equation (3.3):


(3.3) $$T_{s}=0.49\times [T_{d5\%}+0.6\;(T_{d30\%}-T_{d5\%)}],$$

**Table 2.** Thermal properties of VLYS-E, VLEV-E and DDM-E.

sample	*E*′ at 30℃ (MPa)	*T*_g_ (°C)	*E*_r_ (MPa)	*υ*_e_ (mol m^−3^)	*T*_d5%_ (°C)	*T*_d30%_ (°C)	*T*_s_ (°C)
VLYS-E	1745	141	14	1236.3	244.5	400.1	165.55
VLEV-E	2959	171	357	29572.7	282.8	429.5	181.70
DDM-E	1623	132	17	1531.6	375.7	397.0	190.36

where *T*_d5%_ and *T*_d30%_ were temperatures at 5% and 30% weight loss, respectively. The *T*_d5%_ of both VLYS-E and VLEV-E were lower than DDM-E, which was attributed to the methoxy and Schiff base in VLYS-E and VLEV-E, have low bond energies, so early decomposition on thermal exposure [29,30]. However, it is noteworthy that their *T*_d30%_ (440.1 and 429.5°C) were higher than DDM-E (397.0℃), which was because the dense carbon layer generated by the cross-linking of the Schiff base during initial decomposition inhibits further thermal degradation of the substrate. Thus, the *T*_s_ for the two prepared resins, especially VLEV-E at 181.7℃, was slightly lower than DDM-E (190.4℃) (table 2). Compared to other reported epoxy systems containing Schiff base structures, the epoxy vitrimers in this work, especially VLEV-E, integrating highly competitive *T*_s_ and *T*_g_ (figure 5). Figure 4d delineated the DTG curves. It revealed that the maximum decomposition temperatures (*T*_max_) for both VLYS-E and VLEV-E were higher than DDM-E and their corresponding maximum decomposition rates (*R*_max_) were much lower. This indicated a substantial inhibition of thermal decomposition in VLYS-E and VLEV-E. The findings suggested that the epoxy vitrimers developed in this work possess superior thermal properties.

**Figure 5.**
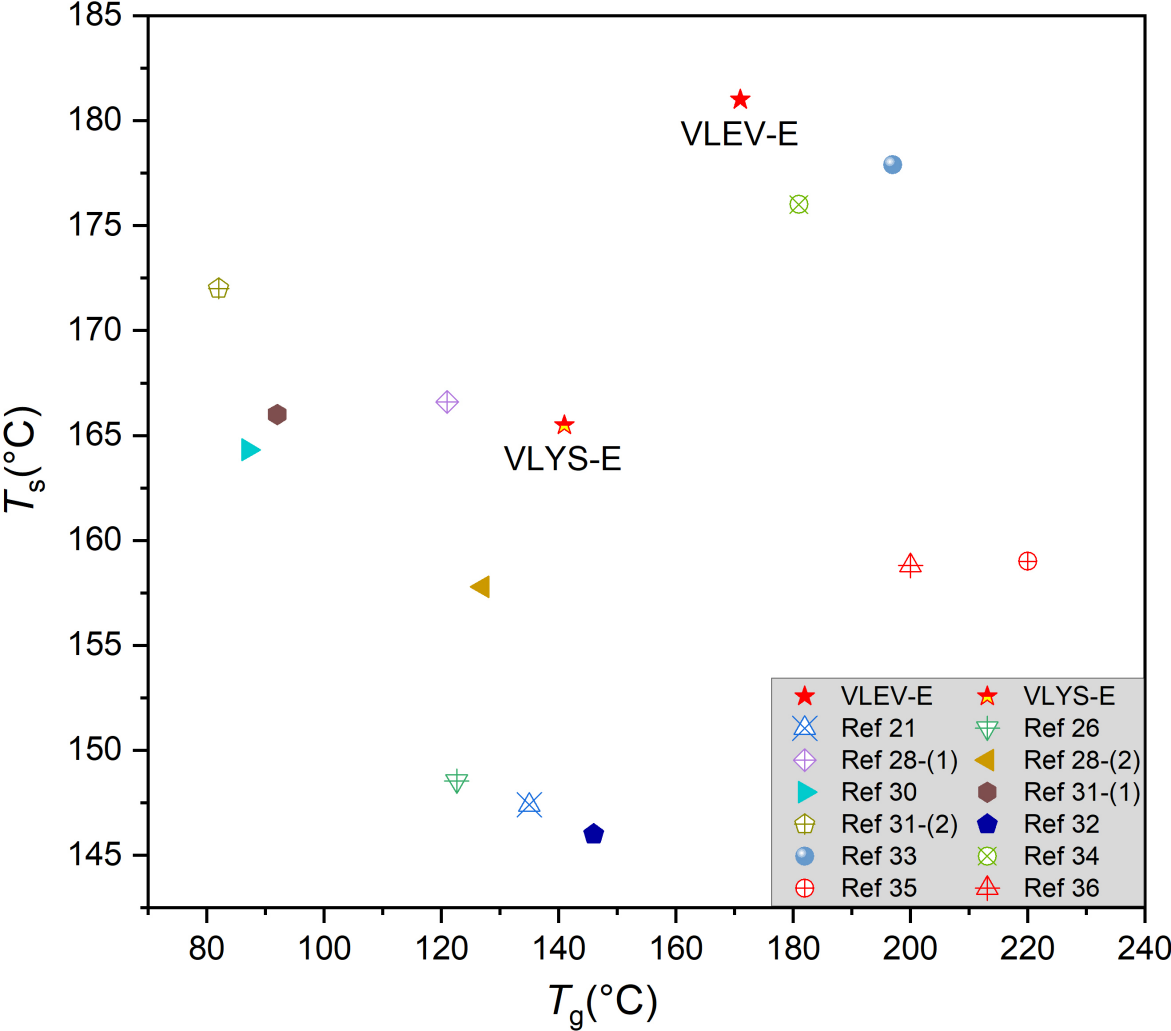
*T*_g_ and *T*_s_ of epoxy vitrimers in this work and literature results [21,26,28,30–36].

### 3.4. Mechanical properties of original and reprocessed resins

**Figure 6.**
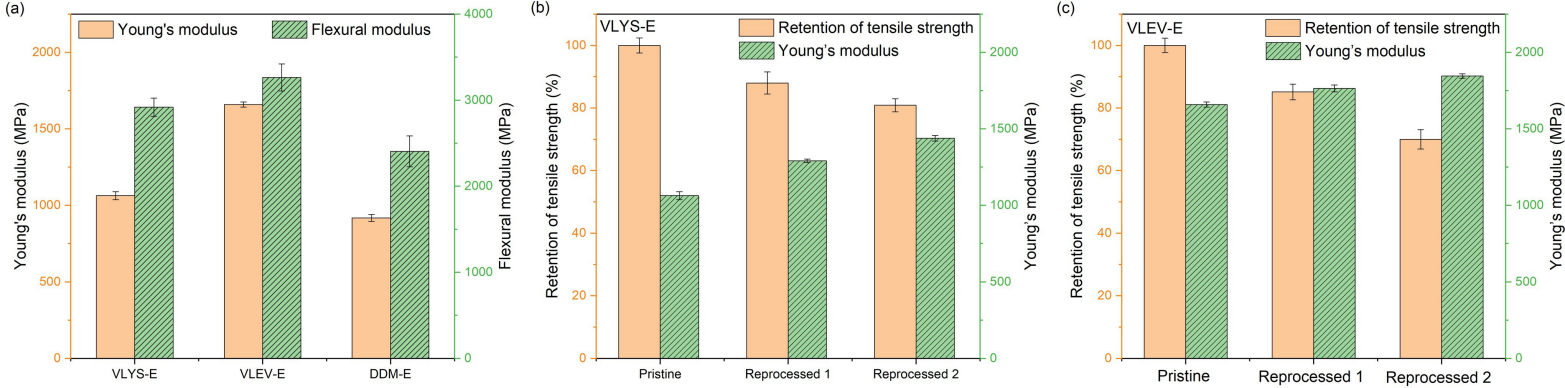
The tensile modulus and bending modulus of VLYS-E, VLEV-E and DDM-E (a), the tensile performance of (b) VLYS-E, and (c) VLEV-E after two cycles.

High-performance epoxy resins are characterized by their combination of superior thermal and mechanical properties. VLYS-E and VLEV-E exhibited higher Young’s modulus and flexural modulus compared to DDM-E. Specifically, the Young’s modulus and flexural modulus of VLEV-E reached impressive levels of 1659.0 and 3265 MPa, respectively, marking 81% and 36% increase over the corresponding values for DDM-E (figure 6a). These figures suggested that the crosslinked networks of VLYS-E and VLEV-E possessed greater structural rigidity, and when subjected to forces of equal magnitude, they demonstrated enhanced resistance to deformation. This aligns with the finding presented in the preceding section. To evaluate the reprocessable nature of the epoxy vitrimers, the cured products were ground into fine powder and subsequently moulded in a steel die. The resin powders of VLYS-E and VLEV-E were reprocessed into new consistency resins after hot pressing. After two reprocessing cycles, the Young’s modulus of the systems increased with the number of cycles (figure 6b,c) because the hot-pressing treatment can be regarded as a post-curing process, leading to an increase in the degree of cross-linking within the reprocessed epoxy resin. The tensile strength of the reprocessed epoxy vitrimers decreased with each cycle, a result of accumulated defects, thermal ageing during the hot-pressing treatment, and the irreversible breakage of non-dynamic bonds during the grinding process. Despite these reductions, the tensile strength retention of the vitrimers after two cycles still exceeded 65%. Flexural testing (electronic supplementary material, figure S3) revealed analogous trends to tensile behaviour. The flexural modulus of VLYS-E and VLEV-E increased from initial values of 2.9 GPa and 3.3 to 3.2 GPa and 3.5 GPa, respectively, after two cycles. More importantly, flexural strength demonstrated exceptional retention stability, preserving above 70% for both formulations after two cycles. By contrast, DDM-E was unable to be reprocessed owing to the absence of dynamic covalent bonds, highlighting the advantage of the vitrimers in terms of reprocessable properties.

### 3.5. Solvent resistance and degradability studies of resins

**Figure 7.**
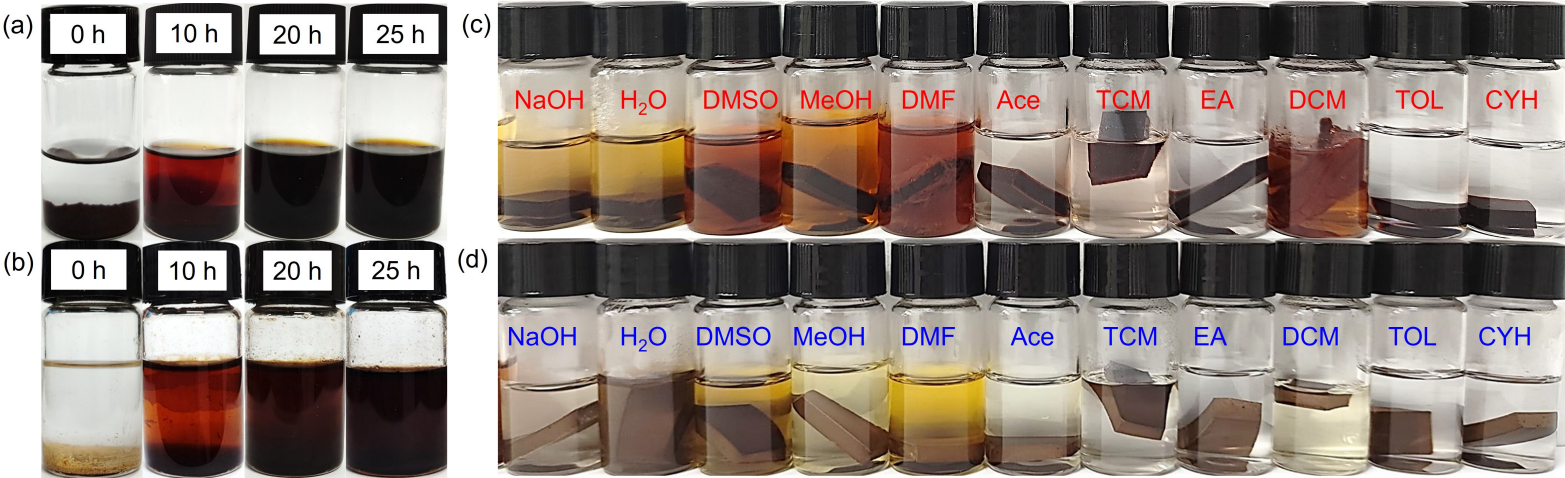
Degradation process of VLYS-E (a) and VLEV-E (b) at different times in 1 M HCl solution (H_2_O/THF (v/v) = 2 : 8) at 50℃. Photographs of VLYS-E (c) and VLEV-E (d) in different solvents for 144 h at room temperature.

The presence of dynamically reversible covalent bonds allowed cross-linked thermosets to degrade under specific conditions, addressing the cyclical challenges faced by conventional thermosets [37]. When subjected to a 1 M HCl solution (H_2_O/THF (Tetrahydrofuran) (v/v) = 2 : 8) at 50°C, which facilitates the swelling and hydrolysis of the resin owing to the addition of THF, VLYS-E was found to be completely degradable within 20 h (figure 7a). VLEV-E, which possesses a significantly higher cross-linking density, can also total hydrolysis within 25 h (figure 7b). Both VLYS-E and VLEV-E had demonstrated satisfactory degradation properties. To reveal the efficient degradation mechanism of VLYS-E and VLEV-E, their hydrolysed products were obtained by removing the solvent from the degradation solution via rotary evaporation, and then analysed by FT-IR (electronic supplementary material, figure S4). After degradation, the characteristic absorption peak of the Schiff base at 1640 cm^−1^ disappeared for original resins, indicating the cleavage of all imine bonds. The new significant absorption peak at 1740 cm^−1^ corresponds to the aldehyde groups hydrolysed by the Schiff base.

Typically, materials that incorporate imine groups often exhibit reduced stability as they pursue degradability. Therefore, we conducted an investigation into the chemical stability of VLYS-E and VLEV-E across a spectrum of chemical environments. Small samples of these two epoxy resins were immersed in various solvents. The solvents were chosen based on their decreasing polarity: NaOH (1 M), water (H_2_O), dimethylsulfoxide (DMSO, methanol (MeOH), dimethylformamide (DMF), acetone (Ace), chloroform (CHCl_3_), ethyl acetate (EA), dichloromethane (DCM), toluene (TOL) and cyclohexane (CYH). The rate at which polymers degrade was generally influenced by two primary factors: swelling and chemical degradation [38,39]. Notably, dichloromethane (DCM has a pronounced swelling effect on epoxy resins that contain aliphatic amines, leading to significant degradation of VLYS-E after 144 h at room temperature (figure 7c) [40]. Despite this, VLYS-E demonstrated robust corrosion resistance, with degradation of less than 3% in all other solvents (electronic supplementary material, table S3). VLEV-E maintained its appearance after 144 h of immersion in a variety of solvents (figure 7d), with degradation rates below 1%. This adaptability was primarily attributed to its highly cross-linked structure, which effectively prevents swelling and degradation in solvents, thereby conferring VLEV-E with outstanding solvent resistance [41].

## 4. Conclusions

In summary, l-lysine and levodopa were selected as the amine components, while vanillin served as the aldehyde source. Through imine condensation reactions, we successfully synthesized two fully bio-based epoxy curing agents featuring Schiff base structures (VLYE and VLEV). The epoxy systems based on these two hardeners, namely VLYE-E and VLEV-E, demonstrated superior thermomechanical properties, with *T*_g_ reaching 141 and 171℃, respectively, surpassing that of the comparative resin system (*T*_g, DDM-E_ = 132℃). Mechanically, VLYS-E and VLEV-E exhibited enhanced Young’s modulus and flexural modulus, with VLEV-E particularly standing out at 1659.0 and 3265.0 MPa, marking an 81% and 36% increase over the comparative sample (DDM-E). Moreover, these bio-based resins were found to be renewable, retaining 65% of their mechanical properties after two cycles. Additionally, both resins exhibited acid degradation capabilities and displayed excellent solvent resistance in non-acidic environments. We believe that this research offers a novel approach to the synthesis of fully bio-based curing agents, which could significantly advance the field of eco-friendly and high-performance recyclable epoxy resins. Furthermore, the research of epoxy monomers based on amino acids and vanillin was taking place in our laboratory.

## Data Availability

The data that support the findings of this study are available in the electronic supplementary material [42].
